# Hydroxypropyl Cellulose/Pluronic-Based Composite Hydrogels as Biodegradable Mucoadhesive Scaffolds for Tissue Engineering

**DOI:** 10.3390/gels8080519

**Published:** 2022-08-19

**Authors:** Daniela Filip, Doina Macocinschi, Mirela-Fernanda Zaltariov, Bianca-Iulia Ciubotaru, Alexandra Bargan, Cristian-Dragos Varganici, Ana-Lavinia Vasiliu, Dragos Peptanariu, Mihaela Balan-Porcarasu, Mihaela-Madalina Timofte-Zorila

**Affiliations:** 1 Laboratory of Physical Chemistry of Polymers, “Petru Poni” Institute of Macromolecular Chemistry, Aleea Gr. Ghica Voda 41 A, 700487 Iasi, Romania; 2 Department of Inorganic Polymers, “Petru Poni” Institute of Macromolecular Chemistry, Aleea Gr. Ghica Voda 41 A, 700487 Iasi, Romania; 3 Centre of Advanced Research in Bionanoconjugates and Biopolymers, “Petru Poni” Institute of Macromolecular Chemistry, Aleea Gr. Ghica Voda 41 A, 700487 Iasi, Romania; 4 Laboratory of Functional Polymers, “Petru Poni” Institute of Macromolecular Chemistry, Aleea Gr. Ghica Voda 41 A, 700487 Iasi, Romania; 5 Laboratory of Polycondensation and Thermostable Polymers, “Petru Poni” Institute of Macromolecular Chemistry, Aleea Gr. Ghica Voda 41 A, 700487 Iasi, Romania; 6 Saint Spiridon County Hospital, Bulevardul Independentei 1, “Gr. T. Popa” University of Medicine and Pharmacy, 16 Universitatii Street, 700115 Iasi, Romania

**Keywords:** hydroxypropyl cellulose, polymeric blends, dynamic vapor sorption, biocompatibility, mucoadhesive gels, tissue engineering

## Abstract

Recently, the development of new materials with the desired characteristics for functional tissue engineering, ensuring tissue architecture and supporting cellular growth, has gained significant attention. Hydrogels, which possess similar properties to natural cellular matrixes, being able to repair or replace biological tissues and support the healing process through cellular proliferation and viability, are a challenge when designing tissue scaffolds. This paper provides new insights into hydrogel-based polymeric blends (hydroxypropyl cellulose/Pluronic F68), aiming to evaluate the contributions of both components in the development of new tissue scaffolds. In order to study the interactions within the hydrogel blends, FTIR and ^1^HNMR spectroscopies were used. The porosity and the behavior in moisture medium were highlighted by SEM and DVS analyses. The biodegradability of the hydrogel blends was studied in a simulated biological medium. The hydrogel composition was determinant for the scaffold behavior: the HPC component was found to have a great influence on the BET and GAB areas, on the monolayer values estimated from sorption–desorption isotherms and on mucoadhesivity on small intestine mucosa, while the Pluronic F68 component improved the thermal stability. All blends were also found to have good mechanical strength and increased biocompatibility on the NHDF cell line. Based on their particular compositions and increased mucoadhesivity on small intestine mucosa, these polymeric blends could be effective in the repair or recovery of damaged cell membranes (due to the contribution of Pluronic F68) or in control drug-delivery intestinal formulations.

## 1. Introduction

Hydrogels represent hydrophilic three-dimensional networks held together by either chemical or physical bonds. For biomedical hydrogels, two of the most important objectives are the choice of compatible and therapeutically acceptable excipients and the preparation of a hydrogel base with a semi-solid consistency, jelly-like structure and bioadhesive properties [1]. Hydrogel scaffolds are structurally similar to the extracellular matrixes of many tissues and can be used for drug and growth factor delivery, engineering of tissue replacements [2] or to replace and improve the functionality and integrity of a tissue, either in vivo or in vitro. Most applications concern cartilage or bone substituents, but also skin, ligaments and organs (bladder, liver, etc.). Such hydrogels must support the attachment of certain cells and, later, ensure their proliferation and differentiation to facilitate tissue growth or restore its function. In other tissue applications, hydrogels are used for surface modification of tissue growth in such a way as to promote or adjust the interaction with the natural body constituents [3].

The tailored properties of hydrogels for tissue engineering should ensure both biocompatibility and biodegradability, as well as mechanical strength and the utilization of the network porosity toward the encapsulation and release of drugs, growth factors and extracellular matrix constituents (proteins, glycoconjugates, glycosaminoglycans, phospholipids, cholesterol, cations/anions, etc.) [4].

In particular, the use of composite hydrogels or hydrogel blends is an original approach with many advantages, allowing different natural/synthetic constituents to be combined and consolidating the properties of each component, thus improving either the mechanical strength or the cell adhesion, or even increasing the biocompatibility or biodegradability in a desired manner. Usually, the polymer scaffolds are well-tolerated when applied in vivo and the immune response cascade is activated, multiple strategies being applied, such as the use of a “stealth” polymer component (polyethylene glycol derivatives) or incorporation of natural polymers favoring a good exchange with the cellular medium. Moreover, the use of intermolecular crosslinks in hydrogel scaffolds can ensure a gradual degradation, so that the side products can be eliminated by natural excretion simultaneously with the adhesion of cells, based on the recognition process mediated by these interactions [5].

Traditional adhesive polymers (cellulose derivatives—carboxymethyl cellulose, methyl cellulose, hydroxypropyl cellulose, poly(acrylic acid) sodium alginate—and chitosan derivatives) are known for their roles in tissue repair, being highly biocompatible and capable of remodeling damage tissue [6].

Hydroxypropyl cellulose (HPC) is a hydrophilic, non-ionic ether of cellulose where some of the hydroxyl groups of cellulose have been hydroxypropylated, forming OCH_2_CH(OH)CH_3_ groups.

HPC have gained increasing interest for pharmaceutical applications. This biocompatible, biodegradable and bioadhesive polymer forms reversible hydrogels with unique characteristics, such as transparency, high water content, spreadability and good skin flexibility [7]. Moreover, the use of cellulose derivatives as biocompatible materials has gained a primary role in the biomedical field due to their physical and mechanical properties, as well as their low toxicity and biodegradability. Such materials are used in healthcare or as novel biomedical products for wound-healing bone and tissue engineering applications, lenses or drug delivery systems [8].

Pluronic is a synthetic polymer made of amphiphilic copolymers consisting of units of polyethylene oxide and polypropylene oxide that also possesses favorable properties, such as non-toxicity, biocompatibility and biodegradability. These synthetic derivatives are used for the solubilization and stabilization of different pharmaceutical excipients, showing low toxicity. Due to their self-assembly ability, Pluronic derivatives/polaxamers are involved in the design of carriers and smart delivery systems for insoluble drugs, ensuring a targeted release through different routes of administration based on their associative and adsorption properties, thus facilitating permeation through lipid bilayers of cell membrane [9,10]. Pluronic is also used in wound-healing (for bone, as a carrier for osseous graft materials) and burn-healing processes in combination with epidermal growth factor (EGF), mimicking the common skin functions and acting as an “artificial skin”. In combination with other polymers (poly(acrylic acid)), it has been proved that it can easily form bioadhesive gels under normal conditions in the body. Pluronic also possesses a specific role in multi-drug-resistance sensitization and can be a good vehicle for sustained release of bioactive compounds, serving as a rate-controlling barrier [11] and a support for the development of bioactive hybrid hydrogel scaffolds [12]. Hydroxypropyl cellulose and poloxamer 407 were studied as in situ gel-forming systems for controlled delivery of vancomycin [13]. It was found that increased concentration of Pluronic F68 resulted in increased bioadhesive force in Pluronic gels [14]. Thermosensitive chitosan/Pluronic composite hydrogels were synthesized for use as tissue adhesives and hemostatic materials [15].

In this work, hydroxypropyl cellulose and Pluronic F68 hydrophilic polymers were used to prepare hybrid hydrogel scaffolds by freeze-drying. The resulting blend scaffolds were characterized by means of FT-IR and ^1^H NMR spectroscopy, scanning electron microscopy (SEM), differential scanning calorimetry (DSC), thermogravimetry (TGA) anddynamic vapor sorption (DVS) to investigate the resulting properties as functions of the blend scaffolds’ compositions. In addition, their biocompatibility, as studied on NHDF cells, and mucoadhesivity on porcine mucosa open a perspective for multidisciplinary applications in the field of biomedical engineering.

## 2. Results and Discussion

### 2.1. FT-IR Analysis

**Figure 1:**
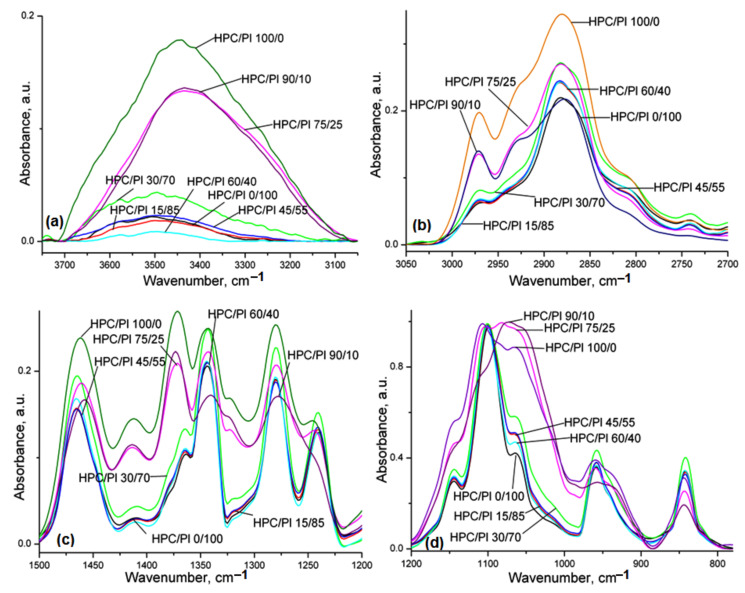
FT-IR spectra of the studied blends: (**a**) 3750–3050 cm^−1^; (**b**) 3050–2700 cm^−1^; (**c**) 1500–1200 cm^−1^; (**d**) 1200–780 cm^−1^.

The FT-IR spectra of the neat HPC, Pluronic F68 (Pl) and the corresponding HPC/Pl hydrogel blend scaffolds are presented in Figure 1. In Figure 1a, the bands located at 3447 and 3507 cm^−1^ are attributable to –OH stretching of the pure HPC and pure Pluronic F68 (Pl) hydrogel scaffold, respectively. For the blend scaffolds with high amounts of HPC (>60%), the –OH peaks were close to those of pure HPC but with lower intensities, and, for the blend scaffolds with low amounts of HPC (<60%), the –OH peaks were close to those of pure Pluronic F68 with various intensities. The bands located at 2971 (peak) and 2925 (sh) cm^−1^ (Figure 1b) were attributed to the asymmetric and symmetric vibrations of the C-H of the methylene groups of the anhydroglucose units. For blend scaffolds with high amounts of HPC, the bands located at 2925 cm^−1^ were still visible, but they vanished for the blends with lower amounts of HPC. The bands located at 2971 cm^−1^ in the blends with high amounts of HPC were close to those of pure HPC but with lower intensities, whereas, for the blends with low amounts of HPC, they were close to those of pure Pluronic with various intensities. The bands located at 1466 cm^−1^ (pure Pl) and 1461 cm^−1^ (pure HPC), attributed to asymmetric bending of CH_3_, were shifted for the HPC/Pl blends. The bands located at 1410 cm^−1^ and 1321 cm^−1^, attributed to O-H and C-H bending, became close to those of pure HPC with higher amounts of HPC in the HPC/Pl blends. The bands located at 1200–1000 cm^−1^, attributed to the C-O stretching mode, shifted and became close to those of pure HPC with higher amounts of HPC in the HPC/Pl blends. The bands located at 958 cm^−1^, attributed to the O-H bending mode, become close to those of HPC for higher amounts of HPC in the blends [16].

The spectral changes recorded for the studied blend scaffolds revealed there were interactions between the two components, which were indicative of some miscibility.

The calculated (with a 50% Lorentzian and 50% Gaussian function) areas of the peaks in these spectral regions (Figure 1) were used to estimate the three regular crystallinity indexes, the total crystallinity index (TCI), the lateral order index (LOI) and the hydrogen bonding intensity (HBI) [7], as the results for the ratio of the area corresponding to the following IR bands: TCI = (A_1374_/A_2920_), LOI = (A_1416_/A_843_) and HBI (%) = (A_3350_/A_1337_). The calculated values of these indices of crystallinity are shown in Table 1.

The presence of the crystalline structure in the cellulose chains was highlighted by the band at 1416 cm^−1^, while the amorphous regions could be estimated by the band at 843 cm^−1^. The C-H bending mode marked by the band at 1374 cm^−1^ and the stretching of C-H bonds observed from the band at 2900 cm^−1^ were useful for the calculation of the total crystallinity in hydrogel blends, while, for estimation of intermolecular organization in blends, the specific absorption bands at 1337 cm^−1^ and 3350 cm^−1^ were used.

We observed a decrease in H-bond energy and distance after the addition of Pluronic to the hydrogel composition. This process was accompanied by an increase in the TCI index with the addition of up to 60% Pluronic followed by its decrease at higher concentration of Pluronic as result of the reorganization of the H-bonding interactions between both components. At huge concentrations of Pluronic, the organization of hydrogel blends led to a major decrease in the LOI and HBI as compared to pure HPC due to the amorphous behavior of Pluronic, which changes the degree of crystallinity of HPC (Table 1) through their interactions. The H-bond energy and distance also highlight this aspect in the form of lower values for E_H_ and slightly increased values for the H-bond distance, supporting the interconnectivity between both components.

### 2.2. ^1^H NMR Analysis

**Figure 2:**
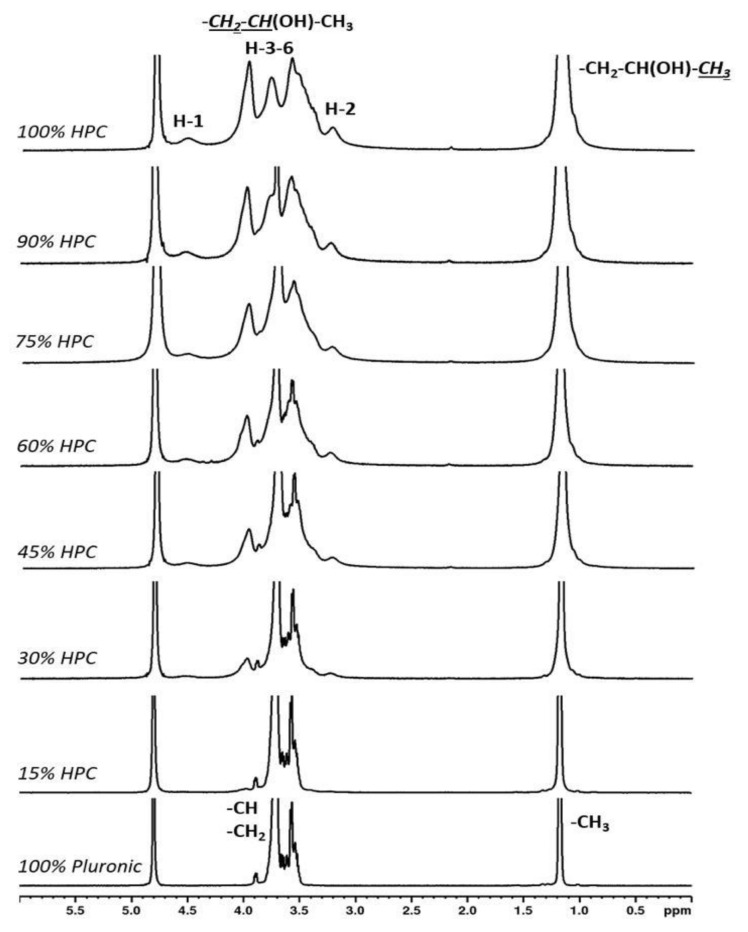
^1^H NMR spectra of hydrogel blends with different additions of HPC in their compositions.

The ^1^H NMR spectra recorded for 100% Pluronic showed peaks for the methyl groups at 1.63–1.79 ppm and peaks for the methylene and methyne groups at 3.52–3.90 ppm. The ^1^H NMR spectra for HPC showed characteristic peaks for the protons from the glucose units of cellulose: the anomeric protons H-1 at 4.52 ppm, H-2 at 3.23 ppm and H3-6 at 3.41–3.97 ppm, which overlapped with the peaks for the methylene and methyne groups of the 2-hydroxypropyl substituent. The methyl group from the 2-hydroxypropyl substituent of cellulose gave a peak at 1.17 ppm. The spectra recorded for the variously proportioned mixtures did not show any chemical shift changes or additional peaks other than the ones from Pluronic and HPC, indicating that the samples were physical mixtures and that no chemical structure changes took place (Figure 2).

### 2.3. SEM Analysis

**Figure 3:**
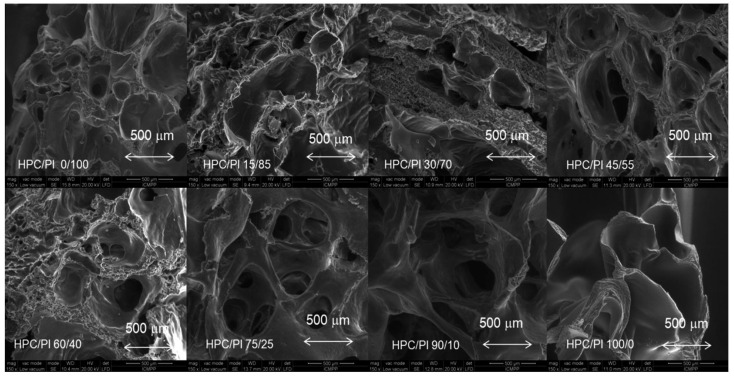
SEM micrographs of the cross-sections of the HPC/Pl blend and pure HPC and Pluronic F68 hydrogel scaffolds.

The morphologies of the HPC/Pluronic F68 (HPC/Pl) hydrogel scaffolds obtained after lyophilization are presented in Figure 3. It is evident from Figure 3 that highly porous structures were obtained after freeze-drying was performed. The pores were a result of ice crystal formation and are similar to those of other natural polymer hydrogel scaffolds [17,18].

A porous architecture is crucial for cells in terms of growth, diffusion of nutrients, oxygen and removal of cell metabolites from the hydrogel scaffold. SEM micrographs of the cross-sections of the HPC/Pl blend matrices showed the presence of interconnected pores of various sizes. With increasing HPC content, the interconnectivity of the pores became enhanced. Interconnected porous structures are favorable for swelling and water uptake.

### 2.4. Dynamic Vapor Sorption Analysis

**Figure 4:**
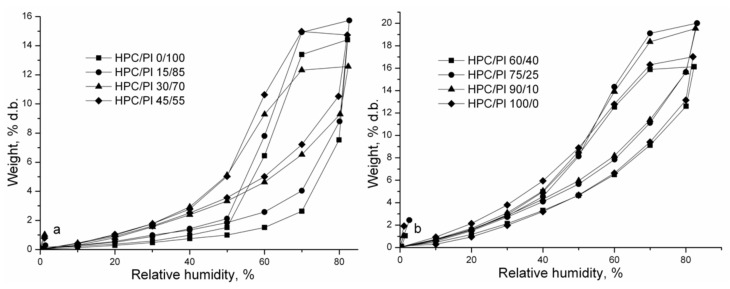
Sorption/desorption isotherms for HPC/Pl scaffolds with <60% HPC *wt*/*wt* (**a**) and (**b**) >60% HPC *wt*/*wt*.

The sorption/desorption isotherms of the studied HPC/Pl scaffolds are shown in Figure 4. The adsorption and desorption isotherms indicate the dependence between the equilibrium water content of the HPC/Pl scaffolds and the relative humidity. Hysteresis behavior is due to water desorption occurring more quickly than water sorption; i.e., the percentage of water loss is higher than that of water gain in the same period and with the same relative humidity. Higher hysteresis with 60–80% relative humidity was obtained for increased amounts of HPC, indicating that the surfaces of these samples had greater polarity than the water molecules, and their uptake was increased.

The values of the diffusion coefficients were calculated by employing Equation (1) and Equation (2), respectively, and they are tabulated in Table 2.

For *M_t_/M_∞_* < 0.5 with short times:

(1) $$\frac{M_{t}}{M_{\infty }}=\frac{4}{l}\cdot \sqrt{\frac{D_{1}\cdot t}{\pi }}\;$$

And (*M_t_*/*M_∞_*)^2^ = 16·*D*_1_·*t*/*π*·*l*^2^ = *K*_1_·*t*, where *K*_1_ = 16·*D*_1_/ *π*·*l*^2^; result: *D*_1_ = *K*_1_*πl*^2^/16.

For *M_t_*/*M_∞_*·> 0.5 with long times:

(2) $$\frac{M_{t}}{M_{\infty }}=1-\frac{8}{\pi^{2}}\cdot e^{-\frac{D_{2}\pi^{2}t}{l^{2}}}$$

And ln(1−*M_t_*/*M_∞_*) = ln8/*π*^2^−*D*_2_·*π*^2^·*t*/*l*^2^ = *K*_2_·*t*, where *K*_2_ = −*D*_2_·*π*^2^*/*l*^2^;* result: *D*_2_ = −*K*_2_*l*^2^/*π*^2^.

In these equations, *t* is the time measured when the concentration is changed; *l* is the plate thickness; *M_o_* is the initial equilibrium mass and *M_t_* = *M_o_*−*M_∞_*, which is the change in the mass from *M_o_* to the new equilibrium mass *M_t_*; and *D* is the diffusion coefficient.

At short times (*Mt/M∞* < 0.5), the values of the diffusion coefficient (*D*_1_) were lower than those (*D*_2_) corresponding to long times (*M_t_*/*M_∞_* > 0.5), as presented in Table 2. *D*_1_ increased with increasing amounts of Pluronic in the blend scaffold because of its hydrophilicity and lower molecular weight. Polymers with low T_g_ have greater segmental mobility and show enhanced diffusivity. *D*_2_ was found to be higher for increased amounts of Pluronic.

BET and GAB mathematical models [19,20,21] were used to fit the experimental data. The monolayer sorption values describe the amount of water that is strongly adsorbed to specific sites at the polymer substrate surface, and they were given by both the BET and GAB models. The surface area of the polymer adsorbent was calculated from the monolayer capacity. The surface area and monolayer capacity value determined by the BET and GAB models are presented in Table 3.

From Table 3, it is evident that the values for the BET area and monolayer were higher for increased amounts of HPC in the blend scaffolds. The values for the GAB area and monolayer increased with increasing amounts of HPC in the blend scaffolds. These results can be correlated with the interconnected pore morphology, which increased with increasing HPC content. The specific surface area of a sample is dependent on the generation of pores in the drying method. When aerogels are prepared from wet samples, the pore structure becomes distorted during the drying process and various changes occur at small scales in the pore structure, leading to a higher specific dry area. There are some reports that found variations in the specific surface after using the drying method for nanofibrillated cellulose or regenerated cellulose with similar values of 400–500 m^2^/g [22].

### 2.5. Mechanical Tests

The mechanical properties of hydrogels were found to be essential for their successful application as drug delivery systems ensuring a controlled release of drugs or as polymeric scaffolds to provide support for natural cell adhesion and tissue growth. Moreover, the designed scaffolds had to accomplish different functions depending on the type of tissue, so there were some particular mechanical requirements. The scaffold stiffness determines different rates of cell proliferation, differentiation and migration. One of the main disadvantages of physically crosslinked hydrogels is the lack of mechanical strength and stability compared to covalently crosslinked ones. However, beyond this disadvantage, the original rigidity of the polymer chains in the structure of the hydrogels and the density of the crosslinking through intermolecular interactions can produce the desired mechanical strength [23].

**Figure 5:**
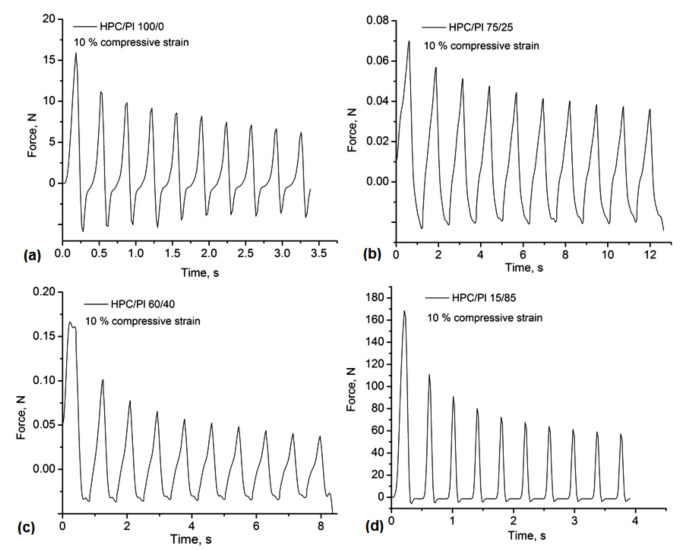
Compression tests of the monoliths under 10% compressive strain: (**a**) HPC/Pl 100/0; (**b**) HPC/Pl 75/25; (**c**) HPC/Pl 60/40; (**d**) HPC/Pl 15/85.

**Figure 6:**
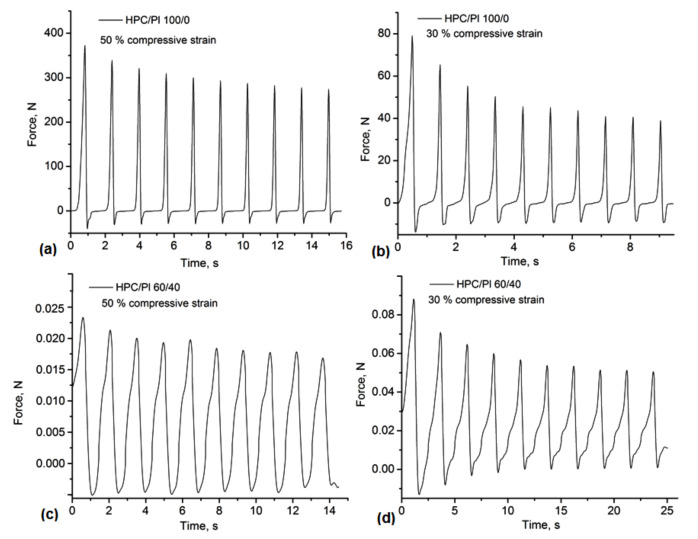
Compression tests of the monoliths under 30% and 50% compressive strains: (**a**) HPC/Pl 100/0—50% strain; (**b**) HPC/Pl 100/0—30% strain; (**c**) HPC/Pl 60/40—50% strain; (**d**) HPC/Pl 60/40—30% strain.

The uniaxial compressive assay was performed in order to evaluate the mechanical stability of the HPC/Pluronic blends. The compressive stress–time curves for different compression rates of 10%, 30% and 50% are shown in Figure 5 and Figure 6. All hydrogel-based blends could be compressed with over 50% strain, with some deformations or fractures associated with the complete release of water from the porous structures. In pure HPC and in hydrogels with higher amounts of Pluronic in the composition (HPC/Pl 15/85), an increase in the network stiffness was observed, highlighted by a rapid decrease in the mechanical strength even at the 10% compression rate (Figure 5). In the stress–time curves for the cycle loading–unloading tests, a greater decrease in the stress was observed, especially under 30% and 50% strain (Figure 6). These results suggested that the hydrogels lost their porous network integrity under compression in a moist medium, similarly to other physically crosslinking hydrogels [24]. However, the behavior of the blends was similar due to the H-bonding dissociation during the hydration, with the exception of the HPC/Pl 75/25 under 10% compression and the HPC/Pl 60/40 under 50% compression, where the decrease in the stress became negligible after the first cycles. Overall, these hydrogel-based blends could offer a good strategy for the development of soft materials with self-healing properties, given the involvement of H-bonding in the network stabilization.

### 2.6. DSC Analysis

**Figure 7:**
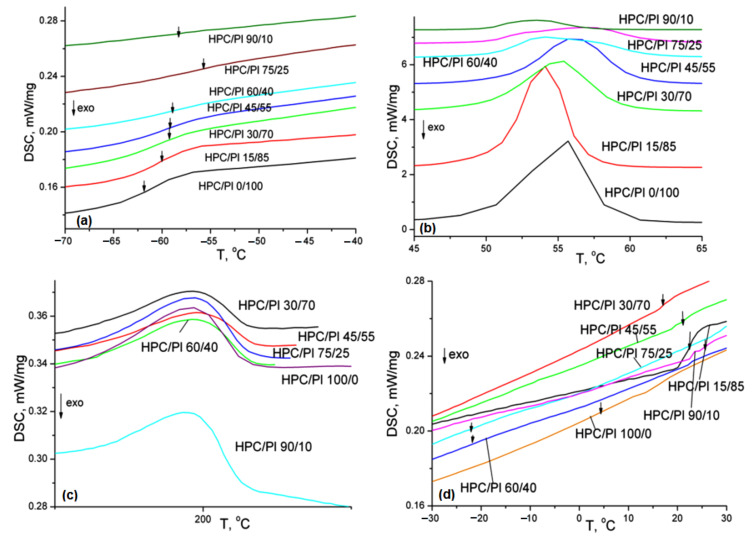
DSC thermograms (second heating runs) for the studied blend scaffolds: (**a**) T_g1-_glass transition temperature corresponding to Pluronic F68 component; (**b**)T_m1_-melting temperature corresponding to Pluronic component; (**c**) T_m2_-melting temperature corresponding to HPC component; (**d**) T_g2_-glass transition temperature corresponding HPC component in the blends.

The DSC thermograms (second heating runs) are depicted in Figure 7. The thermal characteristics obtained from the DSC (second heating runs) with the hydrogel blend scaffolds are tabulated in Table 4.

Based on the DSC data, the weight fraction crystallinity *χ_c_* was determined using Equation (3):

(3) $$\chi_{c}=\frac{\Delta H}{\Delta H_{c}}$$

where Δ*H* is the melting enthalpy obtained from the first heating scan and Δ*H_c_* is the melting enthalpy for a perfect crystallite. For HPC, Δ*H_c_* = 6.44 cal/g [25], while the weight fraction crystallinity *χ_c_* of the powder HPC was found to be 13.6%, which was different from the *χ_c_* of lyophilized HPC, which was found to be 8.2%, proving that the blend mixture obtained through the freeze-dry process changed the interconnectivity in a specific way. This was also proved by the decrease in the TCI index found in the FT-IR spectra after increasing the HPC content in the hydrogel blend composition.

Thermal transitions in the blend scaffolds were revealed by means of DSC thermal analysis. DSC is employed to discriminate between miscible and immiscible polymer blends. A single T_g_ dependent on blend composition is indicative of a miscible system, two T_g_ similar to the related T_g_ of the components are indicative of an immiscible blend and two T_g_ in between the values of the pure components are indicative of partially miscible systems. As shown in Table 4 and Figure 7, two T_g_ values related to those of pure components were obtained. T_g1_ showed values to the close composition, revealing that the phase interactions were weak physical, not chemical, interactions and depended on modifications in the morphology, along with changes in the composition. In contrast, T_g2_ showed different values from the composition. The position of T_g_ and the miscibility of the polymer blends are dependent on the interactions between the components, as well as the free volume [26]. A reduction in T_g2_ is associated with an increase in the free volume [27] and indicates non-compatibility between the two polymers [28]. A change in the Δc_p_ with the composition can be explained by the differences in the Δc_p_ values of the pure components. The decrease in Δc_p_ combined with the composition for the blends showing limited compatibility can be explained by the dissolution of that component in the conjugate phase [29,30].

When a blend exhibits two or more melting temperatures, it is indicative of the immiscibility of the polymers and of a multi-phase material. Two melting points near those of the pure components were obtained. They corresponded to the melting of the two individual components, which were phase-separated. It has been found that, in the case of two completely immiscible polymers, the heat of fusion normalized to the component’s content should remain a constant [31]. The normalized values of the melting enthalpy (Table 4), ΔH_m1n_ and ΔH_m2n_, showed deviations from a constant value, which was indicative of the presence of weak miscibility.

### 2.7. Thermogravimetric Analysis

**Figure 8:**
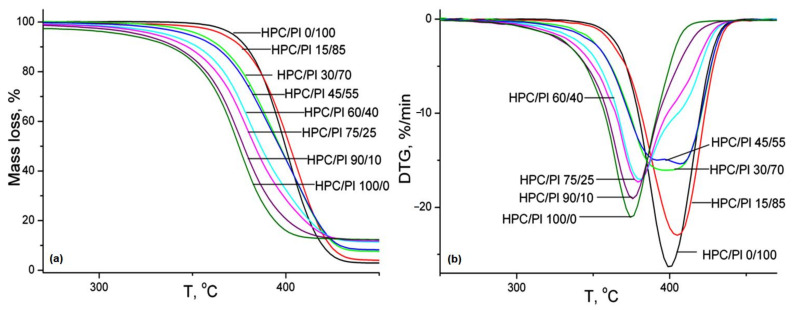
TG-(**a**) and DTG-(**b**) curves for the studied hydrogel blends.

The TG and DTG curves of the HPC/Pl blend scaffolds are presented in Figure 8. The thermal characteristics and kinetic parameters obtained from the TG and DTG curves are tabulated in Table 5. The onset decomposition temperatures of HPC/Pl 0/100 and HPC/Pl 100/0 were 358.8 and 294.4 °C, respectively. It is evident that, with increasing amounts of HPC in the scaffold blends, the onset thermal decomposition temperature decreased and the resulting blends became less thermally stable. The activation energy and order of reaction, as determined by means of the Coats–Redfern method [32], decreased with increasing amounts of HPC, which was also indicative of diminished thermal stability. The weight loss corresponding to the decomposition stage decreased with increasing amounts of HPC because of its higher molecular weight. With higher amounts of Pluronic in the blend scaffolds, the T_max_ values became close to those of neat Pluronic and, with higher amounts of HPC in the blend scaffolds, the T_max_ values shifted toward those of neat HPC. This was evidence of some degree of miscibility between HPC and Pluronic. For completely immiscible blends, the DTG curves were superpositions of the DTG curves of pure components.

**Figure 9:**
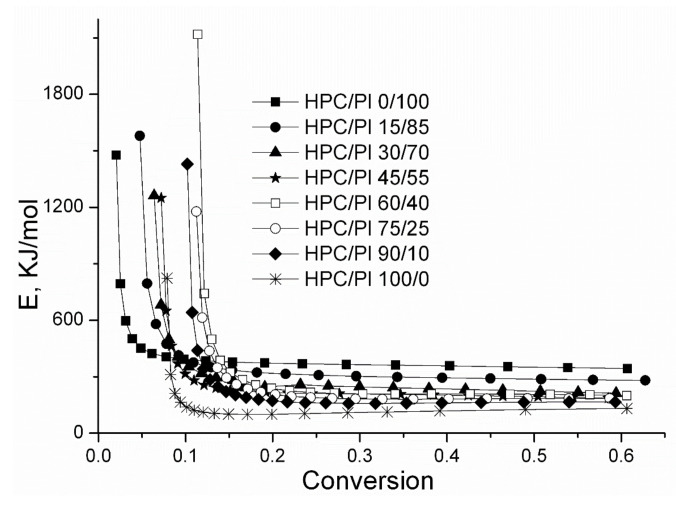
Variations in activation energy as a function of conversion.

The variations in the activation energy as a function of conversion, as determined using the Reich–Levi method [33], for the studied blend scaffolds are presented in Figure 9. At the initial stage of thermal degradation, the abrupt drop in the activation energy occurred in relation to the loss of volatile compounds. Afterwards, the activation energy tended to remain constant and the values maintained the same order as that found with Coats–Redfern method.

### 2.8. Cell Toxicity

Evaluation of the biocompatibility of materials intended for medical applications is the main step before their acceptance in clinical use, as cell involvement is indispensable in tissue regeneration. Cell culture is a facile investigation method that allows the estimation of the cytoxicity of a material during implantation in a biological medium, thus limiting the toxic effects from unwanted side products, which would lead to unwanted damage. Hydrogels are considered ideal materials for tissue engineering, especially due to their similarities with extracellular cell matrix. It has therefore been established that their composition can be designed to provide controlled molecular responses and ensure cell attachment and structural integrity, thus limiting the immune response and inflammation when they are used [23].

Cellulose-based hydrogels are recognized for their good performance in biomedical applications thanks to their adhesive properties. They have been used for wound dressing, drug delivery systems and as pharmaceuticals, proving both their biocompatibility and biodegradability, which can ensure proliferation, cell migration and, also, blood vessel infiltration, enhancing the pharmaceutical efficiency of different drugs [34,35,36].

The cytotoxicity of a biomaterial is dependent on the administered dose, with a higher dose increasing the risk of toxicity in the exposed cells. For wound dressings, fibroblast cells play the main role, especially through their migration into the wound, which is followed by the proliferation stage, when the deposition of extracellular matrix occurs. The designed hydrogel-based blends using HPC and Pluronic F68 can support such cellular processes. While the HPC component ensures the adhesive properties for cells, Pluronic F68 increases the fibroblast growth factor, being itself a good substrate for cell proliferation. Moreover, the inclusion of Pluronic F68 in the hydrogel blends may provide a synergistic effect through specific drug encapsulation or through its own proved ability to inhibit the growth of various leukemia cells [37].

The biocompatibility of the materials was tested by investigating the viability of NHDF cells after they were exposed for 48 hours to the presence of these materials in the culture medium. The CellTiter 96^®^AQueous One Solution Cell Proliferation Assay MTS test is based on the mitochondrial transformation of the MTS reagent into a formazan that absorbs light at 490 nm. The higher the absorbance value is, the more metabolically active the cells are. This test is essentially an indirect means of determining cell viability and proliferation.

**Figure 10:**
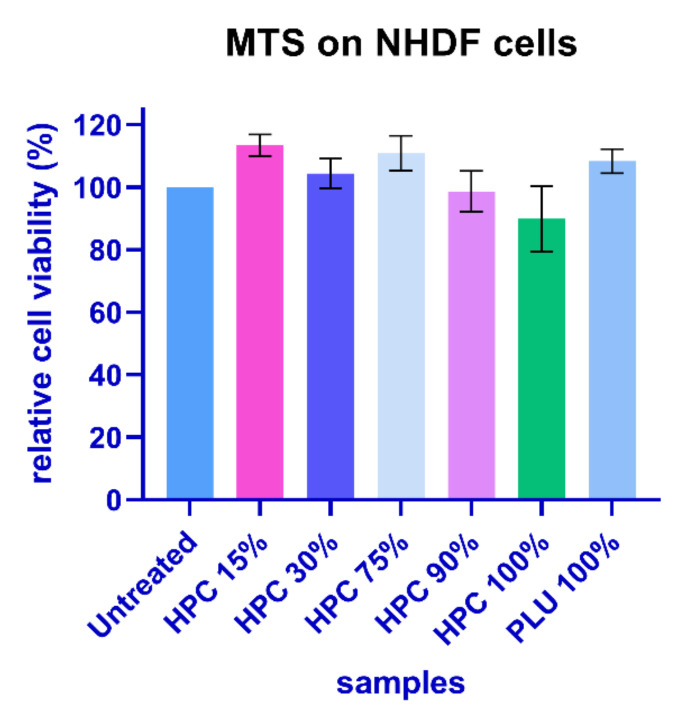
Relative cell viability according to the MTS assay. NHDF cells were cultivated in the presence of test materials for 48 h. The values represent means ± S.E.M., n = 3. *p* < 0.05 (HPC 100%); *p* < 0.01 (HPC 90%, HPC 75%); *p* < 0.001(HPC 30%); *p* < 0.0001 (Pluronic 100%, HPC 15%).

According to this test, the viability of the cells treated with HPC15%–HPC90% materials did not fall below 98%, except in the case of the HPC 100% material, for which the viability decreased to 90% (Figure 10).

However, it should be noted that, from a statistical point of view, these values were not significantly lower than in the case of the control for any of the materials (the *p* value was higher than 0.05 in the one-way ANOVA test).

Thus, we can say that the results of the MTS test verified the premises for good cellular tolerance in all the tested materials, supporting the ability of these gels to be used in tissue engineering applications.

### 2.9. Hydrolytic Stability

**Figure 11:**
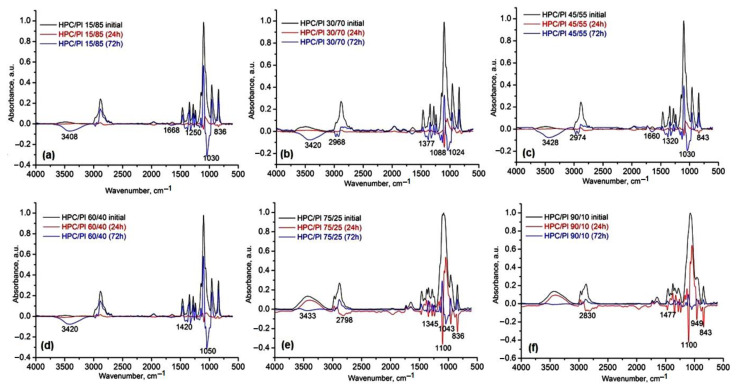
IR subtracted spectra before and after biodegradation of hydrogels in PBS medium at pH 7.4 for: (**a**) HPC/Pl 15/85, (**b**) HPC/Pl 30/70, (**c**) HPC/Pl 45/55, (**d**) HPC/Pl 60/40, (**e**) HPC/Pl 75/25, (**f**) HPC/Pl 90/10.

Taking into account the fact that the HPC/Pluronic blends were designed for tissue engineering applications, we evaluated their degradability in PBS at a physiological pH of 7.4 for a period of 72 h. The degradation products were identified using FTIR spectroscopy. Given their compositions involving physical crosslinks, it was confirmed that, in a first degradation step, the dissociation of the H-bonds occurred, followed by the release into the medium of fragments of two polymer components: HPC and Pluronic. The results of the FT-IR spectroscopy analysis using the subtracting function are shown in Figure 11. The behavior of the hydrogels under physiological conditions differed depending on the hydrogel composition, the ratio between HPC and Pluronic and the intermolecular interaction strength formed by the freeze-drying process: with increased concentrations of HPC in the blends (HPC/Pl 75/25 and HPC/Pl 90/10), very good stability was observed, with the release of some HPC fragments after 24h and of Pluronic after 72h (Figure 11e,f). With lower concentrations of HPC, greater release of HPC was observed after 72h (Figure 11a–d). In all blends, the first product released was generally Pluronic, which could be an advantage for delivery of drugs and other bio-constituents for tissue engineering.

### 2.10. Bioadhesion and Mucoadhesion Tests

**Figure 12:**
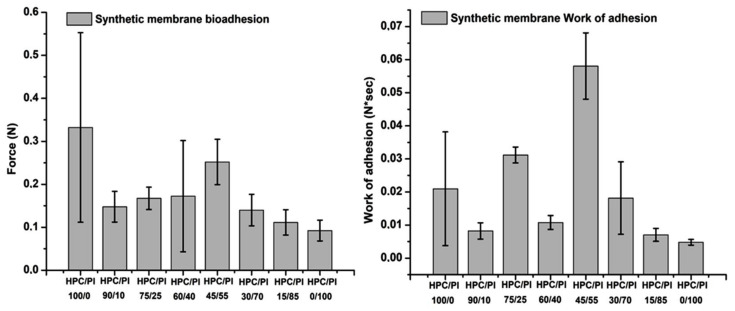
Bioadhesion evaluation of the studied blend scaffolds.

**Figure 13:**
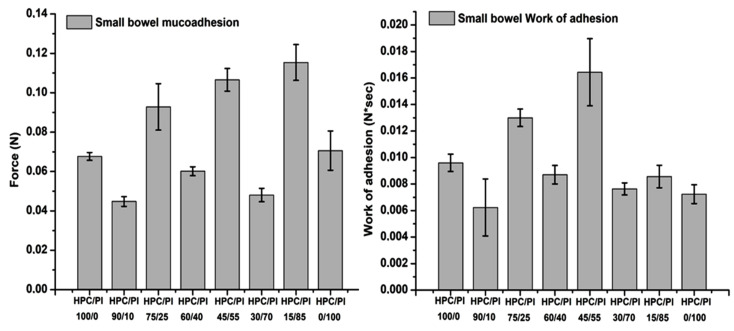
Mucoadhesion evaluation of the studied blend scaffolds.

Adhesive properties in biomaterials are highly sought-after characteristics in pharmaceutical applications of biocompatible polymers, especially hydrogels. Given their mucoadhesive capacities, these composites can act as both hybrid scaffolds and bring therapeutically active agents, if they are incorporated in blends, to the desired sites through multiple routes of administration. Bioadhesion, and adhesion generally, is an interface phenomenon. There is a direct dependence between the ability of the material to form connections with the substrate and the bioadhesivity. Various theories explain the interactions between the two meeting components, including adsorption, mechanical and diffusion theories, all of which lead to the same conclusion: the stronger the connection between the two components, the better the adhesion properties [38]. Cellulose-based materials have been tested in many forms (fibers, films, coatings, etc.) in terms of their adhesion mechanisms for many years now, as they can be used in a wide range of applications in different fields, cellulose being an abundant compound [38,39,40]. Hydrogels can hold water in their structures through the pores formed by the polymer crosslinked chains. This is possible because of their hydrophilic characteristics, a trait that improves the bioavailability of some compounds, especially when loaded with therapeutic active ingredients with weak hydrophilic properties, and because of the interactions on the mucus layer; thanks to its high-water content, mucus itself represents a hydrogel, a biopolymeric layer, making hydrogel scaffold formulations compatible candidates for drug delivery applications [41,42,43]. The afore mentioned HPC blends were evaluated for their bioadhesive performance (Figure 12) on a dialysis cellulose membrane support, as this type of substrate can mimic the biological tissue and the data obtained can be compared to the results gained from the mucoadhesive testing (Figure 13), where a biological mucosa was used as a substrate. The cellulose membrane was boiled for 30 minutes and left to cool before being sampled in the required dimensions and stored in phosphate-buffered solution at pH 7.4. For the mucoadhesion tests, porcine small bowel mucosa was used with a pH 8.2 buffer solution, mimicking the conditions in a biological medium.

With regard to the bioadhesion evaluation of the studied blends (Figure 12), it was observed that the best adhesion force on cellulose synthetic membrane was obtained for the HPC/Pl 45/55 blend. HPC/Pl 100/0 showed a large variation in the registered values due to the higher swelling behavior in the PBS medium. However, higher wettability is not desirable for adhesive support as it reduces the retention period required for repair processes. Lower values were acquired for the adhesion force and the work of adhesion in the high-content Pluronic blends.

The mucoadhesion values for the studied blend scaffolds tested are illustrated in Figure 13. Better mucoadhesion was observed in the small bowel biological membrane, given that, here, movement of the mucin chains also occurred; thus, there was better interaction at the interface with the material. The best detachment (adhesive) force result was obtained with the HPC/Pl 15/85 blend, suggesting that a high content for the Pluronic component contained in the matrix can lead to better mucoadhesion [9,10,11]. Very good adhesion force and work of adhesion were obtained for the HPC/PL 45/55 blend, where the Pluronic component and the compound matrix approached equality, giving a better intermolecular interaction between the blend and the mucosa and suggesting that equilibrium between the blend components can improve the mucoadhesion property.

## 3. Conclusions

In this work, new HPC/Pluronic F68 blend scaffolds were prepared by freeze-drying. FTIR spectroscopy showed that there were interactions between the two components, which were indicative of the miscibility of the hydrogel blends. SEM microphotographs revealed that, with increasing amounts of the HPC component, the interconnectivity of the pores in the blends increased, which was also responsible for the swelling and water uptake. DSC analysis revealed thermal transitions in the individual components, which were indicative of phase separation. The analysis of the change in heat capacity and melting enthalpy revealed that the studied blends were partially miscible. The dynamic vapor sorption analysis showed that the diffusion coefficients increased with increasing amounts of Pluronic. The BET and GAB areas and monolayer values were found to increase with increased HPC contents due to the increased interconnectivity in the pores. The thermal stability increased with the increasing Pluronic content, as evidenced by the variation in the onset temperature of degradation and the values of the activation energy. All samples proved to have excellent compatibility with fibroblast cells, mechanical strength (depending on the blend composition) and water sorption capacity, as well as good hydrolytic stability and bio- and mucoadhesivity with synthetic cellulose membrane and porcine small bowel mucosa. The HPC component led to increased values for the detachment force in interaction with the synthetic membrane, while the Pluronic component led to increased values for both the synthetic membrane and the small bowel mucosa due to its influence on the wettability of the blend scaffolds, as well as the interpenetration and chain diffusion processes occurring during the consolidation step of adhesion. The particular properties of these blend scaffolds, derived from their composition (high water content, biocompatibility, biodegradability), and their increased mucoadhesivity on small intestine mucosa make them suitable biomaterials for tissue engineering applications in the repair or recovery of damaged cell membranes (thanks to the contribution of Pluronic F68) or in control drug-delivery intestinal formulations.

## 4. Materials and Methods

### 4.1. Materials

Hydroxypropyl cellulose (Klucel LF M_w_ 95,000 Da, 99% purity) was purchased from Aqualon, Hercules Inc. (Wilmington, USA), and used as received. Poly(ethylene glycol)-block-poly(propylene glycol)-block-poly(ethylene glycol) (Pluronic F68) was purchased from Sigma-Aldrich USA as PEO_76_PPO_29_PEO_76_ (M_n_ ≅ 8400 g/mol, ≥99% HPLC, with 85% PEO groups and without additives (phenol red)) and used as received. The materials used for the biocompatibility study were: a normal human dermal fibroblast (NHDF) cell line purchased from PromoCell(Heidelberg, Germany); Eagle’s Minimal Essential Medium Alpha (aMEM) and 1% penicillin–streptomycin–amphotericin B mixture (10K/10K/25 µg in 100 mL), purchased from Lonza Verviers, Belgium); fetal bovine serum (FBS), purchased from Biochrom GmbH (Germany); Tryple, purchased from Gibco(Langley, VA, USA); phosphate buffered saline (PBS),purchased from Invitrogen (Eugene, OR, USA);and a CellTiter 96^®^AQueous One Solution Cell Proliferation Assay, purchased from Promega(Madison, WI, USA). All materials intended for cell culture tests were used as received, without additional purification, based on the specifications of use for cell studies.

### 4.2. Preparation of the Blended Aqueous Solutions

**Figure 14:**
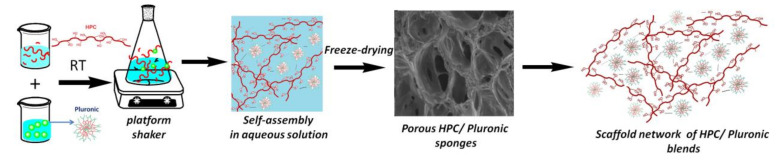
Schematic representation of the preparation of porous scaffold networks (sponges) based on HPC and Pluronic F68 blends. Hydrogen bonds are highlighted withdashed lines in the representations of the scaffold networks of HPC/Pluronic blends.

Aqueous solutions of each polymer were prepared in distilled water. The various blends of HPC and Pluronic F68 were obtained by mixing their aqueous solutions in the required proportions (HPC/Pluronic F68: 15/85; 30/70; 45/55; 60/40; 75/25; 90/10) and then stirring them for several hours over a platform shaker at room temperature. The total concentrations of the blend solutions were kept constant at 25 *w*/*v*. Aqueous solutions of the pure HPC and Pluronic F68 at 25 *w*/*v* were also prepared, and then all solutions were subjected to freeze-drying processes to obtain sponge blend structures with intermolecular crosslinks (Figure 14).

### 4.3. Methods

***Lyophilization*** was performed to prepare the sponge blends, freezing them in liquid nitrogen with the help of freeze-dryer equipment (LABCONCO FreeZone Freeze Dry System) at −50 °C, 1.510 mbar, for 24 h.

***ATR-FTIR*** spectra were measured with a FTIR Bruker Vertex 70 spectrometer possessing a ZnSe crystal. The wavenumber spectral range was set at 4000–600 cm^−1^. Thirty-two scans at a resolution of 4 cm^−1^ were applied to register the spectra at room temperature.

The –OH spectral region in the range 3700–3000 cm^−1^ was deconvoluted with the curve-fitting method using OPUS 6.5 software. The position of the peaks was estimated from the second derivative of the spectra. The area corresponding to each maximum was calculated with a 50% Lorentzian and 50% Gaussian function. Based on the –OH area, the energy and the H bonding distances were calculated based on the Sederholm [44] (Equation (4)) and Struszczyk [45] (Equation (5)):

Δ*ν* (cm^−1^) = 4.43 × 10^3^ × (2.84 − *R*) (4)

where Δ*ν* = *ν* − *ν*_0_, and *ν*_0_ is characteristic of the “free” O-H groups situated at 3650 cm^−1^, while *ν* is specific to the H-bonded wavenumber in the IR spectra of the samples.

(5) $$E_{H}=\frac{1}{k}\frac{\nu_{0}-\nu }{\nu_{0}},$$

where 1/*k* is a constant with the value of 2.625 × 10^2^ kJ.

Based on the IR spectra, the calculated areas of the peaks at 1374 cm^−1^, 2920 cm^−1^, 1416 cm^−1^, 843 cm^−1^, 3350 cm^−1^ and 1337 cm^−1^ were used to determine the degree of self-organization of the samples through the calculation of the three standard crystallinity indexes: total crystallinity index (TCI = A_1374_/A_2920_), lateral order index (LOI = A_1416_/A_904_) and hydrogen bonding intensity (HBI = A_3350_/A_1337_) [7].

***^1^H NMR*** spectra were recorded on a BrukerAvance NEO 400 MHz Spectrometer equipped with a 5mm QNP direct detection probe and z-gradients, using standard parameter sets provided by Bruker. The spectra were recorded in D_2_O at room temperature and were calibrated on the solvent residual peak (4.800 ppm).

***SEM analysis*** was carried out using a Quanta200 scanning electronic microscope with an integrated EDS system and a GenesisXM 2i EDAX with SUTW detector; the magnification is indicated on the micrographs.

***Dynamic vapor sorption (DVS)*** was employed for the determination of water sorption/desorption isotherms. An IGAsorp apparatus (a fully automated gravimetric analyzer supplied by Hiden Analytical (Warrington, UK)) was employed. This equipment is used to investigate water sorption at atmospheric pressure by passing a humidified stream of gas over a sample. The IGAsorp is equipped with a sensitive microbalance (resolution of 1 μg and capacity of 200 mg), which continuously registers the weight of the sample, together with the temperature and relative humidity around the sample. Isothermal studies can be performed as a function of humidity (0–95%) in the temperature range from 5 to 85 °C, with an accuracy of ±1% for 0–90% RH and ±2% for 90–95% RH. The relative humidity (RH) is controlled by wet and dry nitrogen flows around the sample. The RH is held constant until equilibrium or until a given time is exceeded before changing the RH to the next level.

The ***mechanical tests*** were carried out on swollen samples as monoliths, about 50 mm in diameter and 10 mm in length, using an Instron 3365 two-column universal mechanical testing device. All measurements were performed in laboratory conditions at room temperature by using an extension and compression rate of 50 mm/min and different compressive strains: 10%, 30% and 50%. Before the registrations, the compression plates were brought into contact with the materials by applying a force of 0.1 N.

***Cell toxicity*** was studied using normal human dermal fibroblast (NHDF) cells, which were cultured in complete medium prepared from alpha-MEM, supplemented with 10% FBS and 1% penicillin-streptomycin-amphotericin B mixture. The cultures were maintained in a 5% CO_2_ humidified atmosphere at 37 °C and the cells were cultivated until a sufficient amount was obtained for the experiments. For the passage, the medium was removed and the cells were washed with PBS, then detached with Tryple.

For the MTS assay, 25 × 10^5^ cells/well were seeded into 24-well plates and incubated overnight for cell adhesion. The next day, the samples were prepared as follows: 25 mg was weighed from each sample and placed on top of each well previously seeded with cells, and the plates were returned to the incubator.

After 48 h, the plates were treated with MTS reagent according to the manufacturer’s protocol and the obtained formazan was quantified after 2h by measuring absorbance at 490 nm with a plate reader (BMG Labtech, Ortenberg, Germany). The cell viability was estimated as the absorbance of the samples as a percentage of the absorbance of the untreated cells.

***Statistical analysis*** was undertaken by using GraphPad Prism v.9 for Windows (GraphPad Software, La Jolla California USA, www.graphpad.com), accessed on 12 May 2022, to analyze the viability data. The results are shown as means ± the standard error of the mean (S.E.M.), n=3. To compare multiple groups, a one-way ANOVA test was used and the difference was considered significant when *p* < 0.05.

The ***Hydrolytic stability*** of the hydrogel blends was evaluated in vitro in PBS phosphate buffer at pH 7.4. The degree of degradation of the hydrogels was monitored with FT-IR spectroscopy over a period of 72h using the spectra subtraction procedure, a function available in OPUS 6.5 software.

***Bioadhesion and mucoadhesion tests*** were performed using a TA.XTPlus^®^ texture analyzer (Stable Micro Systems, UK) in order to estimate the adhesion of the materials to cellulose membrane and small bowel porcine mucosa. The material samples used were cut to the same dimensions as the cylinder device, and the cellulose membrane was pre-boiled and cooled before being cut into 4 cm^2^ pieces in order to fit the holding device. Over each of the cellulose membrane samples, 100 µL of phosphate buffered saline solution with a pH of 7.4 was added for the simulation of the physiological environment. Furthermore, for the accurate simulation of the physiological environment, the holding device with the cellulose membrane, over which the cylinder with the material was lowered to produce contact, was placed in heated distilled water to 37 °C and stirred with a speed of 250 rotations per minute. The cylindrical device with the material sample was lowered with a pre-determined speed of 1 mm/s to reach the cellulose membrane with a determined contact force of 1 gf and for the pre-determined time of 30 s. The maximum detachment force and the work of the adhesion parameters were determined using the own TA.XT Plus^®^ texture analyzer software.

## Figures and Tables

**Table 1:** Curve-fitting results for the -O-H spectral region, energy and distances of the hydrogen bonds in the studied hydrogels.

Sample	ν _H-bonded–OH_, cm^−1^	E_H_ (kJ)	R (Å)	TCI (A_1374_/A_2920_)	LOI (A_1416_/A_843_)	HBI (A_3350_/A_1337_)
HPC/Pl 100/0	3263	27.832	2.753	0.91	6.36	2.16
HPC/Pl 90/10	3266	27.616	2.752	1.01	3.25	3.65
HPC/Pl 75/25	3320	23.732	2.765	1.44	6.75	1.94
HPC/Pl 60/40	3364	20.568	2.775	2.63	0.52	0.02
HPC/Pl 45/55	3384	19.130	2.779	1.08	0.48	0.3
HPC/Pl 30/70	3390	18.698	2.781	0.88	1.5	0.42
HPC/Pl 15/85	3400	17.979	2.783	0.49	0.46	0.17
HPC/Pl 0/100	3344	22.009	2.771	2.02	0.1	0.05

TCI, total crystallinity index, LOI, lateral order index, HBI, hydrogen bonding intensity.

**Table 2:** Diffusion coefficients determined from experimental data for Pluronic and HPC mixtures.

Sample	*K*_1_ *, *M_t_*/*M_∞_* < 0.5	*K*_2_ *, *M_t_*/*M_∞_* > 0.5	*l* (cm)	*D*_1_ = *K*_1_*πl*^2^/16 (cm^2^/s)	*D*_2_ = −*K*_2_*l*^2^/*π*^2^ (cm^2^/s)
HPC/Pl 0/100	9.51 × 10^−5^	−0.00044776	0.1	1.87 × 10^−7^	4.54 × 10^−7^
HPC/Pl 15/85	8.76 × 10^−5^	−0.00034771	0.1	1.72 × 10^−7^	3.52 × 10^−7^
HPC/Pl 30/70	7.64 × 10^−5^	−0.00047547	0.1	1.50 × 10^−7^	4.82 × 10^−7^
HPC/Pl 45/55	6.37 × 10^−5^	−0.00022294	0.1	1.25 × 10^−7^	2.26 × 10^−7^
HPC/Pl 60/40	3.66 × 10^−5^	−0.00025346	0.1	7.19 × 10^−8^	2.57 × 10^−7^
HPC/Pl 75/25	4.92 × 10^−5^	−0.0002214	0.1	9.66 × 10^−8^	2.24 × 10^−7^
HPC/Pl 90/10	4.43 × 10^−5^	−0.0002308	0.1	8.70 × 10^−8^	2.34 × 10^−7^
HPC/Pl 100/0	4.33 × 10^−5^	−0.00026628	0.1	8.51 × 10^−8^	2.70 × 10^−7^

* *K*_1_ is the slope of the linear regression between (t-t_R_) and (*M_t_/M_∞_)*^2^ for (t-t_R_)≥0 and (*M_t_/M_∞_)*^2^ < 0.2, where t_R_ is the time delay correction of the equilibrium (minus the ratio between the intercept and the slope) for *M_t_/M_∞_* = 0; * *K*_2_ is the slope of the linear regression between t and ln(1 − *M_t_/M_∞_*) for −1.2>ln> −3.0.

**Table 3:** Surface parameter values estimated on the basis of BET and GAB models.

Sample	BET		GAB	
	A (m^2^/g)	MI (g/g)	A (m^2^/g)	MI (g/g)
HPC/Pl 0/100	37.5	0.0106	18	0.0051
HPC/Pl 15/85	81.5	0.0232	28.4	0.0081
HPC/Pl 30/70	198	0.0566	62	0.0176
HPC/Pl 45/55	141	0.0403	83	0.0237
HPC/Pl 60/40	831	0.2368	145	0.0414
HPC/Pl 75/25	361	0.103	166	0.0474
HPC/Pl 90/10	251	0.0715	179	0.0509
HPC/Pl 100/0	528	0.1504	149	0.0423

**Table 4:** Thermal characteristics obtained from the DSC (second heating run) with the hydrogel blend scaffolds.

Sample	T_g1_ (°C)	Δc_p1_ (J/gK)	T_g2_ (°C)	Δc_p2_ (J/gK)	T_m1_ (°C)	ΔH_m1_ (J/g)	ΔH_m1n_ (J/g)	T_m2_ (°C)	ΔH_m2_ (J/g)	ΔH_m2n_ (J/g)
Pluronic F68 powder	−60.4	0.091	-	-	57.7	119.7	-	-	-	-
HPC/Pl 0/100	−61.7	0.138	-	-	55.5	101.3	101.3	-	-	-
HPC/Pl 15/85	−60.4	0.077	22.4	0.119	54.0	96.23	113.2	-	-	-
HPC/Pl 30/70	−59.5	0.057	17.2	0.031	55.1	69.04	98.6	197.9	1.346	4.48
HPC/Pl 45/55	−59.5	0.052	21	0.030	56.1	62.31	113.3	199.2	1.44	3.2
HPC/Pl 60/40	−59.1	0.031	−22.6	0.01	54.0	35.07	87.7	198.0	1.824	3.04
HPC/Pl 75/25	−55.5	0.02	−22.2	0.015	57.0	28.81	115.2	198.1	2.397	3.2
HPC/Pl 90/10	−58.5	0.01	25.2	0.030	53.7	11.35	113.5	197.2	2.484	2.8
HPC/Pl 100/0	-	-	4.2	0.077	-	-	-	197.7	2.444	2.4
HPC powder	-	-	21	0.065	-	-	-	195.8	2.479	-

T_g1_ and Δc_p1_—glass transition temperature and change in heat capacity corresponding to Pluronic F68 component; T_g2_ and Δc_p2_—glass transition temperature and change in heat capacity corresponding HPC component; T_m1_, ΔH_m1_ and ΔH_m1n_—melting temperature, melting enthalpy and melting enthalpy normalized to Pluronic content corresponding to Pluronic component; T_m2_, ΔH_m2_ and ΔH_m2n_—melting temperature, melting enthalpy and melting enthalpy normalized to HPC content corresponding to HPC component.

**Table 5:** Thermal characteristics obtained from the thermogravimetric analysis.

Sample	Stages (°C)	T_max_^a^ (°C)	Δw ^b^ (%)	E_CR_ (kJ/mol)	n
Pluronic F68 powder	I 364.4–427.7	402.1	97.27	311.03	1.2
HPC/Pl 0/100	I 358.8–425	399.8	96.26	318.14	1.2
HPC/Pl 15/85	I 361.6–428.1	405.1	95.63	245.98	1.0
HPC/Pl 30/70	I 349.3–428.3	397.0	91.84	179.52	0.9
HPC/Pl 45/55	I 342.0–427.4	407.1	90.58	148.09	0.7
HPC/Pl 60/40	I 340.4–389.4 II 389.4–424.8	380.4 407	56.17 30.87	143.07	0.9
HPC/Pl 75/25	I 333.5–390.3 II 390.3–424	379.4 408	57.0 24.3	128.05	0.8
HPC/Pl 90/10	I 322.5–411.3	375.8	86.81	108.65	0.4
HPC/Pl 100/0	I 294.4–347.4 II 347.4–396.1	313 374.9	12.13 73.78	77.79	0.0
HPC powder	I 317.8–404.2	374.5	86.36	93.10	0.0

a—T_max_: temperature corresponding to the maximum rate of decomposition; b—Δw: weight loss percentage corresponding to the degradation stage.

## Data Availability

Not applicable.
